# Understanding online exercise provision: a qualitative study of the perspectives of service users, instructors, and strategic leaders

**DOI:** 10.1080/17482631.2026.2727778

**Published:** 2026-09-09

**Authors:** Phillip M. Gray, Phoebe Perham, Ben Andrews

**Affiliations:** a Directorate of Psychology and Sport, University of Salford, Salford, UK; b Beyond Empower, Salford, UK

**Keywords:** Exercise, online, digital, service users, instructors, strategic leaders, qualitative, perceptions

## Abstract

**Purpose:**

Online exercise may reduce barriers to participation by increasing convenience, flexibility, and accessibility. However, few qualitative studies have examined the perspectives of key stakeholders on its role in exercise delivery. This study explored the perceptions and experiences of service users, exercise instructors, and strategic leaders regarding online exercise.

**Methods:**

Focus groups were undertaken with 68 participants from North West England, comprising 50 service users, 10 exercise instructors, and 8 strategic leaders. Data were analyzed using a coding reliability thematic analysis.

**Results:**

Three overarching themes of barriers, benefits, and facilitators emerged. Common barriers included set-up challenges, exercise safety, privacy, security, and digital literacy. Instructor-specific challenges included insurance, music licensing, and monitoring participant engagement. Common benefits included convenience, flexibility, and social interaction. Strategic leaders viewed online exercise as valuable for underserved populations and supporting progression to in-person participation. Accessibility, guidance, and training were common facilitators, while strategic leaders expressed the need for a business case, integration into organizational strategy, collaboration, and partnership.

**Conclusion:**

Online exercise is perceived as a valuable complement to in-person provision. However, effective implementation requires addressing technical, safety, privacy, and organizational challenges. Inclusive design, improving digital literacy, instructor training, organizational strategy, and cross-sector collaboration may facilitate its adoption.

## Introduction

Physical inactivity is a risk factor for early mortality and non-communicable diseases (Department of Health, 2019). In Western countries, physical inactivity is attributed to 9.3% of all-cause, 9.9% of cardiovascular disease, 10.5% of dementia, and 2.6–9.3% of cancer mortality (Katzmarzyk et al., 2022). To reduce this disease burden, adults should engage in 150 min of moderate physical activity or 75 min of vigorous physical activity per week (Department of Health, 2019). However, guideline attainment within the United Kingdom (U.K.) remains suboptimal at 65% among adults (Sport England, 2025). Moreover, attainment rates are lower among certain demographics, such as females (62%), older adults (44%), people with lower socio-economic status (54%), individuals with disabilities (49%), and those living with obesity (57%) (Sport England, 2025).

Moderate-to-vigorous physical activity can be accumulated through instructor-led, in-person exercise. However, for some people, multifaceted barriers to attendance at in-person exercise exist, including access to transportation and facilities, time constraints, neighbourhood safety concerns, stigma, disability, low self-efficacy, and cultural sensitivities (Gray et al., 2026; Gray et al., 2016). The delivery of synchronous (e.g., Zoom or Facebook Live) and asynchronous (e.g., pre-recorded videos on YouTube) online exercise has the potential to address these barriers by providing a convenient, flexible, and accessible means of participation (Cronshaw, 2022; Guo & Fussell, 2022). The COVID-19 pandemic served as a catalyst for providers to migrate their services online. However, the potential of online exercise to function as a permanent and scalable alternative to traditional service provision has been noted (Cronshaw, 2022). This view is supported by evidence indicating that 38% of group exercise participants in England have participated online, either synchronously or asynchronously (Exercise, Move, & Dance UK, 2022). Notably, 56% of people interested in group exercise, but not currently participating, report a willingness to engage with online provision (Exercise, Move, & Dance UK, 2022). A recent review evidenced high levels of attendance and satisfaction with online exercise, alongside improvements in outcomes such as aerobic capacity, strength, and body composition comparable to those associated with in-person provision (Diaz et al., 2024).

Research examining the delivery of online exercise has predominantly adopted quantitative approaches, with studies investigating the prevalence and predictors of use (Mutz et al., 2021; Parker et al., 2021), adherence (Diaz et al., 2024), and its efficacy for improving health and fitness outcomes (Diaz et al., 2024; Lee et al., 2024). Complementing quantitative research, qualitative methodologies can provide rich insights into people’s perceptions of the acceptability, feasibility, barriers, and risks of online exercise. Integration of these perspectives into the design of future online delivery may foster greater satisfaction, uptake, adherence, and enhanced health outcomes (Mager et al., 2022; O’Cathain et al., 2019). To date, several qualitative studies have explored service users’ perceptions of online exercise. Convenience, timesaving, flexibility, access, and enhanced social connections are known as key motivators, whereas privacy concerns, reduced instructor feedback, the impersonal nature, and digital illiteracy are salient barriers (Cronin et al., 2023; Cronshaw, 2022; Gray et al., 2026; Guo & Fussell, 2022; Taveira & Barbosa, 2024). The limited qualitative research examining instructors’ perspectives has identified the convenience, broader reach, and scalability of online exercise as key motivators, whereas technical issues, cognitive demands, the inability to correct exercise technique, and a perceived decreased quality of social interactions are prominent concerns (Gui et al., 2022; Guo & Fussell, 2022; Islam et al., 2024).

Notably, a scarcity of studies has explored the perspectives of strategic leaders, a group that may offer insights into implementation, sustainability, organizational decision-making, and the future role of online or hybrid provision. Within the North West of England, exercise provision is delivered and supported by leisure, community, voluntary, and public sector organizations, involving multiple stakeholders, including strategic leaders, instructors, and service users. Compared with most regions in England, the North West experiences greater levels of health inequalities, deprivation, and physical inactivity (Sport England, 2025; Office for Health Improvement and Disparities, 2026) Moreover, the region has a relatively higher proportion of populations who may experience barriers to online exercise, such as people with disabilities, or people from ethnically minoritized groups (Sport England, 2025; Office for Health Improvement and Disparities, 2026; Office for National Statistics, 2023). Capturing a broader systems-level perspective by simultaneously examining the views of service users, exercise instructors, and strategic leaders may provide a more comprehensive understanding of barriers, facilitators, implementation challenges, and enable the triangulation of findings. Such insights may have practical utility for exercise instructors seeking to develop sustainable online or hybrid delivery models, increase reach, and diversify income streams (UK Active, 2024) as well as for service providers aiming to design accessible, equitable, and scalable online exercise provision. Therefore, the present study aimed to explore service users', instructors', and strategic leaders’ perceptions and experiences of online exercise provision utilizing a qualitative approach.

## Methods

### Ethics

Prior to data collection, ethical approval (ID = 10859) was obtained from the Ethics Committee of the School of Health and Society, University of Salford, and written informed consent was provided by participants.

#### Study design

A qualitative approach, underpinned by pragmatism, was employed to richly capture participants’ perspectives (Silverman, 2013). Pragmatism does not adopt a particular qualitative philosophical stance, but instead values methodological pluralism and flexibility, valuing both objective and subjective knowledge, and the practical application of findings (Dawadi et al., 2021). This approach, therefore, was selected to generate useful findings to inform the future design and implementation of online exercise provision. To ensure explicit, comprehensive, and transparent reporting of findings, the APA Journal Article Reporting Standards for Qualitative Research were adhered to (Levitt et al., 2018).

#### Researcher characteristics

The research team brought different expertise and experiences to the study. P.G. is an academic with a PhD in exercise psychology and is experienced in physical activity, behaviour change, and qualitative research. B.A. has extensive professional and lived experiences of disability and accessibility, including the development and evaluation of physical activity programmes for disabled people. P.P. was an undergraduate student in Nutrition and Exercise as Medicine. These backgrounds may have sensitized the research team to issues surrounding accessibility, inclusion, physical activity participation, and the potential benefits and challenges of online exercise. B.A.’s lived experience of disability and existing professional relationships with some strategic leaders, instructors, and community gatekeepers may have facilitated rapport and trust, but also may have influenced participants’ responses. Several methods to mitigate potential researcher influence are described throughout the procedures section (e.g., inductive analysis, open-ended questions, intercoder agreement, and research team dialogue).

#### Participants

Participants were recruited from the North West of England using purposive sampling. Purposive sampling enabled the identification and selection of individuals with knowledge and experience pertinent to the research question (Palinkas et al., 2015). Three distinct stakeholder groups were recruited: (1) service users; (2) instructors; and (3) strategic leaders. Service users were recruited indirectly via the gatekeepers of community groups contacted through telephone and email. An emphasis was placed on ensuring the inclusivity of underrepresented demographics by purposively recruiting through community groups that represented ethnically minoritized groups, older adults, disabled people (including people who are autistic, blind, visually impaired, deaf, and people with physical and learning disabilities), without applying a predefined recruitment quota. All gatekeepers of the community groups approached agreed to assist with recruitment and distributed the study materials to potential participants, either in person or by email. However, the number of individuals who received these materials, declined, or did not respond was not recorded. Instructors were recruited from local authority public health teams, leisure trusts, and disability-focused organizations involved in the delivery of physical activity programmes. Strategic leaders were recruited from organizations involved in the strategic planning, commissioning, and delivery of sport, physical activity, public health, and leisure services. Both instructors and strategic leaders were contacted directly by the research team by email or telephone, and all who were invited agreed to participate. Inclusion criteria required participants to be aged 18 and over. Individuals were excluded if they refused to consent to the research or were unable to attend a focus group. Potential participants received an information sheet detailing the study aims, confidentiality arrangements, the voluntary nature of participation, and the right to withdraw without consequence. Reasonable adjustments were made, where required, to support participation, including the provision of accessible study materials and the involvement of support persons or interpreters where appropriate.

A total of 68 participants were recruited, comprising 50 service users, 10 instructors, and 8 strategic leaders. Strategic leaders held senior strategic and operational roles in their respective organizations, including two chief executives, a chief operating officer, a managing director, a strategic partnership manager, a public health strategic manager, a head of partnerships, and a physical activity lead. The participant characteristics are presented in Table I. B.A. had established professional relationships with some strategic leaders, instructors, and community gatekeepers through previous work in the physical activity and disability sectors. These relationships facilitated recruitment and access to participants and may have contributed to rapport and trust in the research process, but did not involve managerial or evaluative responsibilities. The participants did not receive any financial incentives or compensation for participation.

**Table I.** Participant characteristics.

	Service users (*n* = 50)	Instructors (*n* = 10)	Strategic leaders (*n* = 8)
Age, mean (SD)	53.34 (7.23)	39.90 (4.47)	36.75 (3.62)
Female, %	62	60	62.5
Disability^a^, %	56	10	12.5
Learning disability, %	20	0	0
Physical disability, %	20	0	0
Visual impairment, %	12	0	0
Hearing impairment, %	8	0	0
Autism, %	8	0	0
Mental health condition, %	2	0	0
Other/unspecified, %	4	0	12.5
Ethnically minoritized, %	14	0	12.5

^a^ Disability categories were not mutually exclusive; participants could report more than one disability characteristic.

#### Procedures

Data were collected through focus groups, which were selected to maximize time efficiency and provide rich data through the snowballing of ideas. Sessions were conducted on one occasion between May and June 2023, either face-to-face or online via Teams or Zoom. Online focus groups offered participants logistical conveniences, enhanced flexibility and access to geographically diverse individuals, and reduced costs (Stewart & Shamdasani, 2017). The focus groups comprised between 4 and 10 participants. Where necessary, support staff and interpreters were also present to facilitate participation. Focus groups were conducted within stakeholder-specific groups (service users, instructors, and strategic leaders) to facilitate discussion among individuals with shared experiences.

Focus groups began with consent for audio recording, introductions, an overview of the research aims, ethical considerations (e.g., confidentiality and the right to withdraw), and ground rules to maximize participant comfort and engagement. Subsequently, an icebreaker activity was undertaken, followed by the semi-structured focus group discussion. The focus group guide comprised 10 questions for service users and instructors and 8 questions for the strategic leaders (see Tables II and III). The guide was flexible, allowing for follow-up probes to elicit further detail and clarification. The questions were designed to be unambiguous, non-leading, and open-ended, thereby encouraging detailed responses (Silverman, 2013). The guide was previously piloted in a separate sample and refined to improve clarity, relevance, and comprehensiveness. The moderators (B.A. and P.P.) facilitated conversation, minimized deviation from the topic, used follow-up probes for further detail and clarification, and highlighted similarities and differences in perspectives to stimulate further discussion. They also enforced ground rules where necessary and monitored participants for signs of distress. Upon completion of the focus group discussion, the participants were afforded an opportunity to reflect and provide additional information, and were thanked for their participation, before the session closed. The focus groups lasted an average of 100 min (range = 76–137 min). Subsequently, participants completed a socio-demographic questionnaire, which captured their age, sex, disability status, and ethnicity.

**Table II.** Focus group schedule for service user and instructor stakeholder groups.

Questions	
1	What platforms do you use to access/deliver* online activity, if any?
2	In your view, what are the benefits of using this/these platform(s) over others?
3	Are there any drawbacks to these platforms that you can see and if so, what?
4	Was there anyone who was hesitant or did not want to access/deliver activity online for any reason? If so, could you explain why?
5	What would have been helpful in getting you to use online platforms for activity?
6	Are you aware of any other platforms that you could use to access/deliver activity online?
7	How do you feel about these platforms?
8	Are there any additional features that would make your preferred platform better?
9	If you could describe your ideal platform to access/deliver online activity from, what would it be like?
10	What would be your absolute must-have feature for online activity and what would you say is the worst?

^*^ The word ‘access’ and ‘deliver’ were used for service users and instructors respectively.

**Table III.** Focus group schedule for the strategic leader stakeholder group.

Questions	
1	What do you think of digital activity?
2	What role do you think digital and online provision plays in physical activity?
3	What barriers do you think people face to participating in digital activity?
4	What would encourage, or help facilitate participants’ engagement with digital platforms, if they chose to do so?
5	What would encourage, or help facilitate the sector’s use of digital platforms, if they chose to do so?
6	If you could describe your ideal platform to deliver online activity from, what would it be like?
7	What would be your absolute must-have feature from a delivery perspective for online activity and what would you say is the worst?
8	Is there anything else you would like to discuss in relation to platforms for physical activity?

#### Data analysis

Focus group recordings were transcribed verbatim, anonymized, and uploaded to NVivo (version 13), which can be accessed via Figshare (https://figshare.com/s/1685f690ea84a27c3fed). A coding reliability thematic analysis was employed (Braun & Clarke, 2022), which involves codebooks, multiple independent coders, and the quantification of coding reliability (Braun & Clarke, 2022).

This approach was selected because its structured and systematic procedures aligned with the study’s pragmatic orientation and practical focus, while enhancing the transparency, replicability, and reliability of coded data (Braun & Clarke, 2021; O’Connor & Joffe, 2020). The approach can help maintain coding standards, facilitate reflection and dialogue within the research team to enhance coding frame specification, and reduce reliance on the interpretation of a single researcher (O’Connor & Joffe, 2020). Specifically, the approach to coding and calculating intercoder agreement described by O’Connor and Joffe (2020) was employed. This involved the research team developing the first draft of a coding frame inductively through data familiarization and subsequent discussions. A codebook comprising a list of codes, labels, definitions, and examples was created. The first coder (B.A.) then applied these codes to all data. The data were segmented by B.A. into meaningful conceptual units, with the researcher determining what constituted conceptually distinct breaks in the transcripts (O’Connor & Joffe, 2020). A cleaned file with the codes removed was shared with a second researcher (P.G.), who independently coded the data using the same segments. Both researchers were trained and experienced in qualitative analysis. Files were merged, and intercoder agreement was calculated for each code, with a minimum threshold of 0.8 deemed acceptable (Lombard et al., 2002). All codes surpassed this threshold (mean = 0.86 ± 0.06), and therefore, no further codebook revisions were required.

## Results

The qualitative analysis yielded three overarching themes and associated subthemes, which are presented in Table IV and in-text. The main themes comprised barriers, benefits, and facilitators to online exercise. Identifiable information has been replaced with pseudonyms.

**Table IV.** Themes and subthemes.

Theme	Subtheme	Stakeholder group(s)	Key findings
1. Barriers	1.1 Common barriers	All groups	Set-up challenges
			Unsafe exercise participation and instruction
		Instructors and service users	Privacy and security
	1.2 Barriers unique to one stakeholder group	Instructors	Music licensing
			Insurance
			Engagement monitoring
2. Benefits	2.1 Common benefits	Service users and instructors	Social interaction and support
			Convenience and flexibility
	2.2 Benefits unique to one stakeholder group	Strategic leaders	Reaching underserved groups
3. Facilitators	3.1 Common facilitators	All groups	Accessibility
			Guidance and training
	3.2 Facilitators unique to one stakeholder group	Strategic leaders	Business case
			Inclusion in strategy
			Collaboration and partnership

### Theme 1: barriers

#### Subtheme 1.1: common barriers

Several barriers, common to two or more stakeholder groups, were discussed, including set-up challenges, safety, privacy, and security concerns.

**Set-up challenges.** Set-up challenges were a pertinent barrier across all stakeholder groups, albeit with different focal points. Service users encountered various barriers to online exercise, including the lack of, or inability to afford, the prerequisite equipment, such as laptops, iPads, or a quality broadband connection. These challenges are illustrated by the following

excerpts:

I don't think every person also has a laptop or an iPad. You might have a phone, but you might not have an iPad or laptop (Sharon, service user).

I remember when we first started Zooming and the thing kept wobbling and kept freezing and that and it was really annoying… I was on a Zoom video on Monday night and it froze a couple of times (Ruth, service user).

Not everybody has got access to laptops and iPads. Sometimes people get on phones. It's a very tiny screen. You can't tell what's going on (Keisha, service user).

Instructors also noted set-up challenges that compromised the quality of sessions, including access to equipment and quality broadband, suboptimal audio quality, and the visual appeal of backdrops. This was articulated by Gemma, Mike, and Rachel:

There were a few concerns from some instructors about technology, because I'm thinking more on the Facebook side of things, we'd have music in the background, but be recording on something. So, people would need to add a phone and a tablet, or a laptop and a tablet; two bits of technology (Gemma, Instructor).

For some people, their quality of connection obviously could not be so good. So, it'd be a lot less frames of someone doing the movements for people to actually follow it and stuff like that…so we're just conscious of quality (Mike, instructor).

It was just empty, vacant backgrounds, and we had echoes and all sorts. So, it was quite low-key (Rachel, instructor).

For strategic leaders, set-up challenges related to securing the required budget and IT infrastructure to facilitate online exercise, as highlighted by Andy and Rebecca:

The one driver that stops us from doing more of that, along with the budgets, is the IT side of things from us as a provider; it stops us from doing more of what we would want to do, and it is a real barrier (Andy, strategic leader).

Similar barriers to what participants will have, providers will have… affordability and also the infrastructure to be able to get the streaming right… that’s going to impact providers as much as receivers (Rebecca, strategic leader).

Even with adequate equipment and broadband, limited digital competence among some service users was perceived as a barrier to accessing online exercise, as illustrated by the following quotations:

Well, not everyone can be technical, are they? So, the older generation and things like that. So, it's just a bit frustrating for us when we couldn't get everybody engaged in it (Carly, instructor).

Somebody kept sending me a Teams...I had a couple of people sent me a Teams invite, and I thought what am I supposed to do with this? And I followed all the instructions, and I just couldn't get through. And the Duo, I think I tried it once and it didn't feel the same as Zoom (Ruth, service user).

For me, it is the confidence around this…so, competence…I think definitely, there is a lot of fear when it comes to what am I downloading? What am I giving my data for? You know, like, is this a scam? Like there's quite a lot associated around that, that needs unpicking (Kelly, strategic leader).

**Unsafe exercise participation and instruction.** A common barrier across all stakeholder groups was ensuring safe exercise delivery with correct form and appropriate intensity, with a lack of instructor feedback a key concern. Instructors were particularly concerned about not being cognizant of the composition of attendees and an inability to pre-screen for contraindications to exercise. These safety concerns are reflected in the following comments:

I do very much believe in pre-screening for safety. And with Facebook, I have no control over who's coming, or who is doing what, because you can't correct anybody if you can't see them, can you? (Claire, instructor).

Doing online yoga and Pilates, for example, which I wouldn't usually do as my preferred exercise choice, you aren't able to be adjusted or have personal attention from a tutor in terms of technique, which has caused me a knee injury (Bev, service user).

All stakeholder groups expressed scepticism about the quality and safety of online content, noting that service users with long-term conditions or disabilities could access synchronous or asynchronous sessions inappropriate to their needs. For example:

I think that's a big thing for disabled people. Those with long-term health conditions. How do they know that it's safe? And actually, what's their limit? And their understanding of that? (Rebecca, strategic leader).

What scares me sometimes is that on TikTok, Insta, and YouTube, there's so much stuff on there, and it's just sometimes you think, “Oh, my God, someone could hurt themselves if they did that.” And that's not right. And I hope people who come to you, then go on YouTube, they know, because I hope I educate people enough to know really what's good and what's not (Kate, instructor).

The only thing that scares me about YouTube and physical activity as an instruction…there is so much stuff on there that you don't know if what you're watching is good or bad, and sometimes I watch some stuff and I'm horrified. People who aren't actually properly experienced and who have qualifications (Maggie, service user).

**Privacy and security.** Privacy and security concerns associated with online exercise were noted by service users and instructors. Instructors highlighted that certain platforms do not facilitate the monitoring or vetting of attendees, raising concerns about discriminatory and derogatory language during sessions and professional reputational risks. Both groups highlighted that limited vetting could facilitate predatory behaviour towards vulnerable participants. These concerns are reflected in the following comments:

Some of the concerns that we had prior to delivering were the fact that some of the platforms couldn't be monitored and you couldn't vet. You could access them, especially with Facebook Live. So, our worry with some people, with severe needs, was who they're going to talk to on the internet? Also, derogatory and discriminatory behaviours and the language being used towards them, which didn't actually come about. It was really good, but that was a real worry for us before going to that platform. We said we’d shut it down, but then obviously it’s already in the public domain…but it'd be tied with our sessions, and that was a big potential risk (Dominic, instructor).

It was really freaky, the other day, because he messaged me, and I can't tell you all the things that he was saying about how wonderful, how gorgeous I was and how interesting I was and all that sort of thing, and I'm thinking, “Where's this come from?” And my friend said, “Oh, it's quite nice messages.”, but I said, “You must be joking.” And I just laughed in the end (Ruth, service user).

Aliya noted that the uncertainty about who was present during live sessions raised concerns among participants, particularly regarding cultural sensitivities:

You're just worried that who's sat in that room, people still had the cameras on, but I think if you hit a certain angle, you don't see who's in that room. And so, I did it for a bit, but then I had to stop because when we, of course, when you do your sports, you want to take your scarf off, to get into your gear (Aliya, service user).

#### Subtheme 1.2: barriers unique to one stakeholder group

Instructor-specific barriers included music licensing and insurance considerations, and difficulties with monitoring participant engagement quality during sessions.

**Music licensing.** As highlighted by Sarah and Gemma, instructors experienced barriers related to the use of music during their sessions:

When we put music on, we get about 15–20 min in, and then Facebook switches off and says, “Because you haven't got a licence, you can't use music.” (Sarah, instructor).

The marketing team just said, “Play this and it should fix the issue (of playing music online).” And it did. So, I think it was just choosing the right music, so that it didn't flag up (Gemma, instructor).

**Insurance.** Insurance requirements were a barrier for some instructors. The instructors indicated that their insurers’ expectation for pre-screening was incompatible with the online delivery of exercise, and some expressed concerns about sessions being recorded and reviewed by their insurance companies. These perspectives are illustrated by the following quotations:

But what we're getting back from insurance companies was for online delivery; it had to be camera on, it had to be pre-screened… those demands made it quite difficult for a lot of instructors because they ended up worried. There was a requirement to record all the sessions and then keep hold of those recordings for several years. And all of these became barriers to actually getting people to be comfortable delivering online, because people were wondering what the comeback could be for them if something went wrong (Claire, instructor).

We had the fact that we don't come under the company's insurance…tends to be self-employed and tends to be under our own insurance for our instructors. Because of that, every insurance company has a slightly different approach to online delivery, but the vast majority of them want full pre-screening done and then want written things back from GPs (Carly, instructor).

**Engagement monitoring.** Finally, instructors highlighted their frustration regarding an inability to monitor the quality of engagement from service users during online exercise sessions. Notable comments included:

When we did Facebook, you'd find that you've got 1000 plus views. I mean, I know if it's something like 15 s, they'd have watched it, and it comes up as a view. I'm not sure what it is. So how many of them watch it fully, and how many have just clicked on it, you don't know. But it was coming up with high numbers every time (Sarah, instructor).

It's quite difficult to work out exactly how many views you've had, but we know we did get quite good feedback (Rachel, instructor).

### Theme 2: benefits

#### Subtheme 2.1: common benefits

Several common benefits were reported by two or more stakeholder groups, including social interaction and support, and the convenience and flexibility of online exercise.

**Social interaction and support.** Online exercise was valued by service users and most instructors for its capacity to facilitate social interaction and support. Comments included:

But I think for me, one of the most important things about these things is the interaction. Maybe it's because I live on my own, that might be something, but it's the interaction (Dorris, service user).

It’s just the interaction through Zoom, our members being able to see each other's faces. And I just think it really keeps people going…so, the way it allows people to interact with each other (Carly, instructor).

Some instructors reported difficulties facilitating social interaction, whereas others intentionally allocated time after sessions to encourage service user interaction. For example:

If you were to say, “Just give us two minutes, talk amongst yourselves.”, the chances are, nobody would unmute their mic and say anything, even though we all know we can all have a conversation with each other…awkwardness, slightly, around that feeling of being online. If you're in a room, somebody probably would turn to somebody and say, “Oh, how's it going?” But on a call, it doesn't always feel that way (Fatima, instructor).

We stayed for about 10 min after, and sometimes it went over a little bit because we used to do that in the face-to-face sessions. We would have a 10-minute...we would have tea and coffee at the end of the class and things like that. So, people did enjoy staying for that (Carly, instructor).

**Convenience and flexibility.** Convenience and flexibility emerged as a benefit among the service user and instructor stakeholder groups, particularly with regard to asynchronous sessions. They emphasized the value of offering a variety of pre-recorded sessions, enabling the choice of exercise mode, time, and the ability to leave and return to a session as needed. This was illustrated by the following participant comments:

But I think having a variation of different activities, so it can be around exercise, it can be around relaxation, breathing techniques, posture, all of that's mentioned today, across the week so that people can dip in and out of it (Lorna, service user).

The beauty of being able to have all those videos be saved, so for my company, I've got over 2,000 videos that anyone can access at any time. Like Shaun just said, you could go back 12 months…they're all there for you, so that you can just go on anytime (Carly, instructor).

Service users, such as Bronagh and Ruth, highlighted viewing location flexibility as a benefit of online exercise, particularly for individuals with mobility issues:

The Zoom has been brilliant. These exercises have been brilliant for me because I could never get to all these places where these people go (Bronagh, service user).

But in terms of fitness, yes, this is great. And the fact that I can do it in my dining room as opposed to getting on a bus and going off to somewhere... because I live in Stockport and everything is in Trafford or further afield...so, for those people who have mobility difficulties, it's brilliant (Ruth, service user).

Instructors also highlighted the personal convenience and flexibility that online sessions afforded, as highlighted by the following examples:

I know quite a few people who still do online stuff, and they enjoyed it. It worked better. Who maybe would do one class when they used to come to a session physically, but because they can do one in the morning and then they pick one off after lunch, they'll do two in a day. So, I guess then it works for them because they're doing more activity (Gemma, instructor).

It's convenient not to have to go somewhere sometimes. Do your exercise at home is the biggest plus (Jean, instructor).

#### Subtheme 2.2: benefits unique to one stakeholder group

Reaching underserved groups was a benefit of online exercise, unique to the strategic leader stakeholder group.

**Reaching underserved groups.** Strategic leaders perceived online exercise to increase physical activity participation among traditionally “underserved” groups, including pregnant women, older people in independent living spaces, and disabled people, as indicated by the following comments:

The girls actually enjoyed, you know, the digital offering that was out there, and I think that can be used as a bridge to get people involved in activity and maybe don't have the confidence or the sorts of mental resilience to get involved in team sports and get involved and go out to gyms, etc. (Franchesca, strategic leader).

So, our strategy is really focused on reaching the groups that are underserved, right…there's a convenience factor with digital, let's acknowledge that. You don't need to travel anywhere, and sort of pop it up at your home and all that sort of stuff, but obviously, there's a trade-off that you don't get all the other benefits. So, I think looking at some of those core audiences, so sort of low-income households or people with disabilities or people from sort of underrepresented sort of communities or, you know, diverse heritage. I think it's exciting, and it will play a part (Kelly, strategic leader).

### Theme 3: facilitators

#### Subtheme 3.1: common facilitators

Several common facilitators emerged across two or more stakeholder groups, including enhanced accessibility, guidance, and training.

**Accessibility.** Accessibility was a common facilitator across all stakeholder groups. This included the need for inclusive online exercise provision for diverse populations, including individuals who are deaf, visually impaired, and non-English-speaking. This is encapsulated by the following examples:

The other reason is because I work with the deaf community as well. Zoom, you've obviously got the subtitles. And even if the automated subtitles are not perfect, they are a whole lot better than nothing. So, from that reason as well, that's the other reason Zoom works really well (Claire, instructor).

Take our website, with a click of a button, you can change the colour of the font, the background, the size of the text, the size of the pictures, it converts into a sort of 100 plus languages at the press of a button (Rebecca, strategic leader).

Screen reader, text-to-speech, and control of the computer and the applications by keyboard are needed (Azam, service user).

Service users also frame accessibility in terms of quick and straightforward access to online platforms, as demonstrated in the following excerpts:

I prefer it when I can press on a link and go straight into it. Because if you ask me to type out the numbers and all the passwords and that, that's when I make mistakes (Jeff, service user).

One click and you’re in. There’s no messing about (Tony, service user).

**Guidance and training.** Guidance and training were facilitators common across all stakeholder groups. Firstly, this was discussed in relation to upskilling service users in navigating online platforms, as exemplified by the following quotations:

…I might be able to log on if there are really clear instructions (Lucy, service user).

…around digital literacy. You know, I'm sort of again, you know, obviously, the 1.5 million people that don't have the internet is a problem. The 11 million people that don't know how to switch on devices, connect to Wi-Fi, or log into accounts is another sort of massive problem that we need to sort of understand, and again, I think I heard someone mention how you link in with your local digital inclusion, or sort of digital skills training options, is a really smart move (Kelly, strategic leader).

Jean and Gemma highlighted the need for guidance to support instructors in the effective delivery of online exercise sessions:

I think it would have been useful to have some tips-and-tricks document. So, what about lighting, for example? What way should you have your phone up? Should you have it in landscape or portrait? It's like tips and tricks. What would you do if this happened? Maybe a best-practice guide (Jean, instructor).

I did the first Facebook Live for Trafford, and then they got a few more instructors on. And so, they actually asked me to just write an information sheet. So just with the really simple things like how to set up, how to go live, what's helpful to do and not do…I don't know if it helps anyone, but I think having all the info does ease those technology concerns, and then you can just focus on the delivery of your class and stuff…I guess maybe instructor meetings as well. Just good communication, I guess, if you're in a team who were doing it, between all of you and stuff (Gemma, instructor).

Training for instructors was also discussed in relation to increasing accessibility, as demonstrated in the following excerpts:

…well, they have touched on extra training and not only like the IT training for the coaches and the instructors, but also the, like, accessibility training that they might need (Tony, strategic leader).

You can contact Zoom and request it as an accessibility feature, and they'll add them on even to free accounts. Immediately, you've then opened things up for people who are hard-of-hearing or deaf, who maybe need that additional. So, just a guide as well to how to make sure that your online delivery is as inclusive as possible (Claire, instructor).

I think it would benefit whoever's leading the group or instructing the group to have training. I'm sure (advocacy group) would provide this, to have the opportunity to have some basic awareness training of visual impairment (Alister, service user).

#### Subtheme 3.2: facilitators unique to one stakeholder group

Several facilitators, unique to the strategic leader stakeholder group, emerged. To enable investment in online exercise, strategic leaders needed a robust business case, inclusion in local and national strategy, and collaboration with other organizations to leverage expertise and share risk.

**Business case.** Strategic leaders indicated that investment in online exercise needed a robust business case, which was perceived to be lacking, as described by Rory and Andy:

Ultimately, though, from my role in my organization, it comes down to a proven business case for it (Rory, strategic leader).

The business case and how that would stack up as part of their membership would be, you know, would be one of the biggest considerations for us (Andy, strategic leader).

**Inclusion in strategy.** The inclusion of online exercise within local and national strategy was perceived to facilitate its adoption, maximize impact, and relieve associated concerns. This was highlighted by Rebecca and Fred:

So, we're about to launch the new Greater Manchester Active Strategy in the next couple of months. And we've got digital highlighted specifically (Rebecca, strategic leader).

Manchester has got a plan. They've got a strategy. What I've learned from across the region that I work in, which is the Northwest is, we've got 23 local authorities. You know, it's a real mixed bag, they don't have a plan…if you don't have a plan and a strategy, how can you start to factor in this sort of digital aspect of physical activity of what we're talking about? …So, it's kind of like it goes back to something much more strategic maybe nationally, local, what's the support available for local areas to develop a plan? (Fred, strategic leader).

**Collaboration and partnership.** It was perceived that online exercise delivery could be optimized, and any financial risk reduced, through partnership, collaborative delivery, and investment, as expressed by Rory and Andy:

This is probably where collaboration comes in, in an ideal world and where Amy touched upon aspirations that we've got to improve our digital offer collectively across Greater Manchester. There's maybe something there where we all contribute something into a Greater Manchester offer (Rory, strategic leader).

If there was something that had been produced nationally that we felt assured was quality and just needed us as operators to push that out (Andy, strategic leader).

## Discussion

The current study aimed to explore service users’, instructors’, and strategic leaders’ perceptions and experiences of online exercise provision utilizing a qualitative approach. Salient barriers, common across two or more stakeholder groups, included set-up challenges, the potential for unsafe exercise instruction and participation, and privacy and security concerns. Barriers, unique to instructors, included music licensing and insurance considerations, and an inability to monitor engagement. Salient benefits of online exercise, common to two or more stakeholder groups, included its convenience and flexibility, and the social interaction afforded, whereas reaching underserved groups was a unique benefit for the strategic leader stakeholder group. Accessibility, guidance, and training were facilitators common to all stakeholder groups, whereas facilitators, unique to the strategic leader stakeholder group, included the need for a business case, inclusion in strategy, and collaboration and partnership.

### Barriers

Participants, across all stakeholder groups, highlighted set-up challenges. For service users, this primarily pertained to access to the prerequisite equipment and reliable internet connection for participation, with prior research suggesting this disproportionately affects low-income and rural populations (Islam et al., 2024). Screen freezes, audio lags, and buffering are pertinent frustrations (Cronin et al., 2023; Islam et al., 2024) that undermine service users’ motivation for participation (Islam et al., 2024). Where access to hardware and quality broadband is sufficient, service user participation may be curtailed due to low digital literacy, as suggested by the findings of the current and prior research (Cronin et al., 2023; Islam et al., 2024). Instructors’ specific set-up challenges included the establishment and maintenance of quality audio and visuals. Visually, instructors frequently lack specialized digital infrastructure, such as professional lighting or camera coverage to adequately capture full-body demonstrations (Guo & Fussell, 2022). Moreover, audio quality issues, such as lags or background noise, can disrupt the synchronization of music and movement (Gui et al., 2022; Guo & Fussell, 2022), with some instructors refraining from new or high-intensity movements to avoid confusion (Gui et al., 2022). Thus, given the cognitive demands of the dual role of instruction and production, many instructors perceive the online environment as stressful (Guo & Fussell, 2022). Moreover, the current findings suggest that set-up challenges extend to organizational constraints such as budget and IT infrastructure, alongside platform-specific restrictions, such as the need for music licensing or lack of feedback on viewing figures and engagement quality. These novel findings suggest that online exercise delivery is not only shaped by the availability of prerequisite hardware, but additionally, legal and platform-governance conditions through which sessions are delivered.

The potential for unsafe exercise instruction and participation emerged as a prominent barrier across all stakeholder groups. A distinctive finding is that instructors reported a lack of control within large-scale or open online environments, often being unaware of the quantity and composition of attendees, including an inability to screen for contraindications to exercise. This is exacerbated by some platforms (e.g., Instagram Live), which provide only limited opportunities for meaningful two-way interaction and participant monitoring (Gui et al., 2022). The finding that instructors were unable to monitor and correct techniques has previously been reported (Cronin et al., 2023; Füzéki et al., 2022), with a 90.5% reduction in instructor feedback evident during live-streamed sessions compared to in-person sessions (Islam et al., 2026). Instructors’ frequent reliance on small video thumbnails, low-resolution or fixed camera angles, or no video at all, hinders the accurate assessment and correction of service users’ form (Islam et al., 2026; Guo & Fussell, 2022; Islam et al., 2024). Instructors find it disruptive to move between exercise positions and their device, further limiting real-time feedback (Guo & Fussell, 2022; Islam et al., 2024). Notably, instructors cannot observe posture, form, facial expressions, nor gauge intensity from multiple angles, nor make physical corrections as they would in person (Gui et al., 2022; Guo & Fussell, 2022). This lack of feedback is a key apprehension among service users (Cronin et al., 2023) due to the potential for overexertion and injury risk (Valeriani et al., 2023), and instructors often correspondingly adopt a conservative approach to exercise delivery (Gui et al., 2022). Thus, the insurance requirements identified by instructors in the current study, including participant camera use, pre-screening, and written GP clearance, appear difficult to reconcile with online delivery.

The present findings extend the existing literature by suggesting that perceptions of safety are also precipitated by scepticism regarding the quality of online content. All the stakeholder groups questioned the efficacy, safety, and scientific rigour of online content, often delivered by individuals without relevant vocational qualifications. This was considered salient for those with disabilities or long-term conditions, who may unknowingly access content inappropriate to their needs, abilities, or contraindications. Instructor qualifications, training, and evidence-based practices are key factors influencing service users’ selection of online content (Dennehy et al., 2024). However, the presence of such indicators may not fully mitigate the risk of misinformation or advice being delivered outside someone’s scope of expertise, placing an onus on the service user to critically evaluate online content, despite many not possessing the prerequisite ability or motivation to do so (Dennehy et al., 2024). The safety concerns identified in the current study appear inconsistent with previous evidence indicating that 90% of service users were satisfied with the safety of online exercise (Cronin et al., 2023), alongside reports of low rates of, and mostly mild, adverse events (Diaz et al., 2024). These studies, however, examined structured, synchronous online exercise, which was led by physiotherapists, exercise physiologists, or qualified exercise instructors. In contrast, the participants of the present study additionally discussed open-access and asynchronous content, where opportunities for screening, supervision, and two-way interaction are limited or absent, and expressed uncertainty regarding the credentials of those delivering sessions. Differences in participant characteristics may also contribute, given the high proportion of service users of the current study reporting a disability (56%) for whom concerns over exercise suitability and supervision may be particularly salient.

Instructors and service users perceived significant privacy and security challenges associated with the online environment, including deficient awareness of who is present, inadequate vetting, unwanted contact, and exposure of the home environment. Previous literature highlights service users’ concerns about sharing personal space with strangers (Guo & Fussell, 2022), protecting the privacy of others in their household (Gui et al., 2022; Newbold et al., 2021), exposing a messy home environment, and self-presentation (Gui et al., 2022), which can be heightened by the potential for sessions to be recorded and shared. Consequently, service users frequently disable their cameras and audio (Islam et al., 2026; Guo & Fussell, 2022; Islam et al., 2024), thereby heightening safety risks. The current findings extend the existing literature by suggesting that privacy and security concerns also encompass safeguarding and reputational risks. The potential for predatory behaviour and discriminatory and derogatory language, particularly in open-access online sessions, where instructors have limited ability to vet attendees or intervene before inappropriate comments or behaviours become visible to other participants, was noted. Features of digital interaction, such as reduced identifiability, limited eye contact, psychological distance, and reduced immediate accountability, can increase the likelihood of hostile or abusive behaviour compared with face-to-face encounters (Lapidot-Lefler & Barak, 2012; Suler, 2004).

### Benefits

The convenience and flexibility afforded by online exercise, especially asynchronous modes, were key benefits for both instructors and service users. Consistent with prior research (Cronin et al., 2023; Diaz et al., 2024; Guo & Fussell, 2022; Islam et al., 2024), this pertained to reduced travel requirements and enhanced temporal flexibility and accessibility. The current findings advance this evidence by suggesting that convenience also encompasses greater choice in exercise modes, and the capacity to pause and resume sessions, according to individual circumstances. The ability to pause and resume sessions may be particularly advantageous for individuals with long-term conditions who experience fluctuating symptoms or fatigue (Whitehead et al., 2016) and those with caregiving responsibilities (Ingram et al., 2021). Beyond these pragmatic benefits, this functionality may also support motor learning by enabling participants to repeatedly observe and practice instructor demonstrations at a self-regulated pace (Schmidt & Lee, 2020).

Social interaction and support emerged as a salient benefit of online exercise for service users and most instructors, aligning with the findings of Cronshaw (2022). Nevertheless, consistent with the perceptions of some instructors of the current study, the wider literature predominantly reports reduced opportunities for informal conversations and rapport building, relative to in-person provision (Cronin et al., 2023; Diaz et al., 2024; Guo & Fussell, 2022). This may stem from concerns about conversation privacy (Guo & Fussell, 2022), the inability to facilitate concurrent conversations (Gui et al., 2022), the disabling of cameras and microphones (Islam et al., 2024), and the tendency for people to join immediately before, and depart immediately after sessions (Guo & Fussell, 2022). One explanation for these contrasting findings is that several instructors in the current study were intentional in building connections, as evidenced by their choice of platforms that facilitated reciprocal interaction, such as Zoom, and the allocation of time after sessions for informal interactions, approaches that may enhance social connectedness (Cronin et al., 2023; Diaz et al., 2024; Guo & Fussell, 2022). Moreover, social interaction potentially assumed greater importance for the sizeable proportion of service users with disabilities in the present sample, who are at increased risk of social isolation due to environmental and social barriers (Gómez-Zúñiga et al., 2023).

Strategic leaders highlighted the value of online exercise for addressing inequalities in physical activity participation by extending opportunities to populations underserved by in-person provision. For these populations, online exercise was perceived as a transitional pathway to in-person participation by enabling people with psychological barriers to engage in a less intimidating environment. Prior research indicates that online exercise can attenuate social and physique anxiety, allowing individuals to build self-efficacy in a private, non-judgemental setting (Cronshaw, 2022; Gui et al., 2022; Yoon et al., 2023). However, underserved populations that may benefit most from online exercise, such as those from low-income, ethnically minoritized or rural communities, or those living with a disability, may simultaneously encounter greater barriers to accessing it. Therefore, online exercise should be considered as a potential means of reducing inequalities in exercise participation, contingent on strategies to mitigate digital exclusion, such as digital-skills support, improved access to devices and connectivity, and accessible platform design.

### Facilitators

Accessibility was a common facilitator across all stakeholder groups. Features such as screen readers, text-to-speech, keyboard navigation, and automated subtitles were deemed essential for inclusive service user participation in online exercise (Bercaru & Popescu, 2024). Although automated subtitles were viewed positively, their limitations for deaf users, particularly the use of overly complex language, remain a recognized barrier (Chan et al., 2026). The participants’ preference for one-click access reflects previous findings that passwords and multi-step authentication present barriers for individuals with cognitive and mobility impairments (Tsatsou, 2020). Similarly, preferences for customizable features such as font size, background colour, and language support reinforce recommendations for flexible, personalized technologies that accommodate intra-disability diversity and align with universal design principles (Borg et al., 2015).

The need for guidance and training to increase service users’ and instructors’ knowledge, skills, and confidence was identified as a key facilitator for online exercise. Consistent with previous research (Islam et al., 2024), the participants in the current study identified limited digital competence among some service users. Moreover, the online environment poses an array of technical challenges for instructors (Guo & Fussell, 2022; Islam et al., 2024), with fewer than half of the U.K. fitness and leisure workforce considering themselves as digitally skilled (UK Active, 2024). Consequently, tailored guidance for service users and instructors, regarding platform access, set-up (e.g., camera, audio, and space considerations), troubleshooting, safety, and privacy would be beneficial (Islam et al., 2024). Moreover, the need for instructor-specific training in explaining, demonstrating, supervising, and correcting exercise technique remotely has previously been noted (Diaz et al., 2024). In addition, guidance for instructors on other issues identified in the current study, such as music licensing, accessibility, and insurance, would be beneficial. Digital literacy could be fostered through community-based digital learning hubs, leveraging support from family and peers to facilitate technology adoption (Cronin et al., 2023; Islam et al., 2024), and promoting the use of existing guidance, such as that provided by the Chartered Institute for the Management of Sport and Physical Activity (CIMSPA) (CIMSPA, 2020).

The facilitators identified at the strategic level suggest that the implementation of online exercise depends on organizational conditions that extend beyond service delivery, including integration within organizational strategy, the development of a business case, and inter-organizational collaboration. These findings are supported by the Digital Futures Report (UK Active, 2024), which highlights that only 18% of organizations have an up-to-date digital strategy, while over one-third lack any digital strategy (UK Active, 2024). Moreover, the report highlights ongoing challenges in demonstrating the value of digital investment and identifies collaboration as a key mechanism for improving digital maturity through shared expertise, resources, and learning (UK Active, 2024).

## A synthesis of the distinctive contributions of the present study

Beyond corroborating the barriers, benefits, and facilitators of online exercise previously identified within the literature (Cronin et al., 2023; Guo & Fussell, 2022; Islam et al., 2024), the current study provides several distinct contributions. The simultaneous inclusion of the perspectives of service users, instructors, and strategic leaders provides a more comprehensive understanding of online exercise delivery and participation. The findings advance the understanding of instructor-specific barriers by identifying insurance, music licensing, and difficulties in assessing meaningful engagement as pertinent concerns. Safety concerns extended beyond reduced instructor feedback to encompass uncertainty about qualifications, content quality, and appropriateness, particularly within open-access and asynchronous environments, and for people living with disabilities. Moreover, safety concerns extended to instructors’ lack of awareness of the composition of attendees within open-access environments. Concerns regarding predatory and discriminatory behaviour, and organizational reputational risk, provide additional insight into the privacy and security challenges of online exercise. The enhanced choice of exercise mode afforded by asynchronous online exercise, coupled with the capacity to pause and resume content as needed, extends the understanding of known convenience-related benefits such as reduced travel and temporal flexibility. The current findings also suggest that for the social benefits of online exercise to be maximized, instructors should be more intentional in facilitating such opportunities. The need for a business case, inclusion in strategy, and collaboration provides novel insights into the organizational determinants of online exercise delivery. The findings also advance the understanding of the role of online exercise for underserved populations, including its potential to provide a pathway to in-person participation, while also highlighting the need to mitigate digital exclusion.

### Theoretical interpretation

Collectively, the findings can be interpreted through a Self-Determination theory lens (Deci & Ryan, 1985). The theory posits that people have three basic needs for autonomy (the need for choice and self-direction), competence (the need to feel capable), and relatedness (the need for connection and belonging). The theory, which has substantial empirical support within the exercise domain (Manninen et al., 2022; Teixeira et al., 2012), suggests that when these needs are satisfied, self-determined motivation (e.g., identified or integrated regulation), positive health behaviours, and well-being are likely to ensue. The present study suggests that regarding online exercise, autonomy may be constrained by set-up challenges, such as access to the prerequisite equipment, but supported through a choice of exercise mode, timing, viewing location, and the ability to leave and return to sessions. The need for competence may be undermined by a lack of digital skills, reduced instructor feedback about technique, and an inability to judge safe or appropriate online content, but may be promoted through increased guidance, training, and accessible platform features. Finally, relatedness may be promoted through social interaction and deliberate opportunities for informal interactions, but undermined by the disabling of cameras and microphones, or service users joining immediately before and leaving immediately after sessions.

### Implications

The present findings have implications for service users, strategic leaders, and instructors, which are presented according to the stakeholder group best placed to enact them.

The findings have several implications for service users. Firstly, this includes being cognizant of the salient barriers to online exercise identified in the current study. Where set-up-related challenges arise, service users should seek appropriate support, such as accessing necessary hardware through a digital lending library. Service users concerned about the potential for unsafe exercise participation and instruction may benefit from opting for synchronous, supervised sessions with two-way interaction, including camera use, where appropriate and acceptable. Moreover, prior to participation and through discussions with the provider, they should ascertain the suitability of sessions to their needs and the credentials of those delivering them. Service users concerned about privacy and safety should review the features of available platforms, select one appropriate to their needs before participation, and utilize available privacy and security controls appropriately. Secondly, service users could maximize the benefits of the online exercise identified in the current study. For example, where social interaction is valued, participants could engage in synchronous exercise, select platforms that facilitate communication with instructors and other participants, and select sessions where opportunities for informal interactions are facilitated. Finally, the facilitators identified in the current study imply that service users may benefit from reviewing and selecting platforms that provide accessible features appropriate to their needs, and by availing themselves of guidance and digital-skills support if required.

These findings also have implications for strategic leaders involved in the planning, commissioning, and delivery of online exercises. To mitigate identified barriers, strategic leaders should ensure adequate investment in digital infrastructure, equipment, and connectivity, select user-friendly platforms, offer clear set-up guidance, and signpost service users to device lending or digital skills services where needed. Privacy, security, and safeguarding concerns could be addressed by selecting platforms that support features such as waiting rooms, password-protected sessions, participant vetting, and reporting mechanisms. Safety concerns could be addressed by establishing organizational standards for online delivery, such as instructor competence or pre-screening requirements. Moreover, providers should leverage the benefits of online exercise by offering flexible synchronous and asynchronous delivery modes, opting for platforms that facilitate social interaction, and using online provision to improve access for populations who experience barriers to in-person exercise. This approach, however, would require the concurrent adoption of methods to address digital exclusion. Finally, strategic leaders should consider the facilitators identified in the present study. This may include choosing platforms with accessible features, providing digital literacy support for service users, delivering training for instructors in online delivery, and developing a business case, organizational strategy, and partnerships to support the sustainable implementation of online exercise.

For instructors, particularly independent professionals who retain control over platform choice and delivery decisions, the aforementioned strategic leader recommendations may be relevant. In addition, instructors should be cognizant of the delivery-related barriers identified in the current study. Safety concerns may be mitigated by maximizing opportunities to observe service users, ensuring that demonstrations are clearly visible and audible, and remotely monitoring exercise intensity, for example, through eliciting ratings of perceived exertion. Instructors could also explain the rationale for camera use and normalize its use where appropriate, while also respecting service users’ rights to privacy. The technical aspects of delivery, including camera positioning, lighting, and demonstration space, should be considered and checked before delivery. Moreover, relevant music licensing and insurance requirements should be clarified before delivery. To maximize the benefits of the online exercise identified in the current study, instructors should provide opportunities for informal social interactions before and/or after sessions. Finally, instructors may benefit from available guidance and training in online instruction, technical considerations, and accessibility (CIMSPA., 2020).

### Limitations and strengths

The relatively high proportion of service users living with a disability may have influenced the salience of issues relating to accessibility and inclusion. Experiences were not systematically compared by disability type, and therefore, the findings should not be interpreted as representative of a particular disability group. Moreover, the sample comprised individuals residing in the North West of England, where relatively high levels of deprivation, disability, health inequalities, and physical inactivity exist compared to other English regions (Sport England, 2025; Office for Health Improvement and Disparities, 2026; Office for National Statistics, 2023). Therefore, these contextual characteristics may have influenced the prominence of the barriers, benefits, and facilitators of online exercise identified in the current study. Accordingly, readers should make context-specific judgements on the transferability of findings to populations within other regions. The sample was predominantly female and of White ethnicity, which should also be considered when interpreting the transferability of findings. Although purposive sampling is widely accepted in qualitative research (Shenton, 2004), volunteer bias cannot be ruled out, as individuals with a greater interest in physical activity or online delivery may have been more willing to participate. Despite the benefits of focus groups, the discussion of sensitive or dissenting, yet important, topics may have been constrained (Silverman, 2013). Moreover, the study did not incorporate member checks, prolonged engagement, and data triangulation via observational techniques, which may have further enhanced the trustworthiness of the findings (Shenton, 2004).

Despite these limitations, the study possessed considerable strengths. The inclusion of three key stakeholder groups: service users, instructors, and strategic leaders, is novel and enables the triangulation of perspectives (Shenton, 2004). The purposive recruitment of disabled people, older adults, and ethnically minoritized communities ensured the inclusion of the perspectives of underserved populations in online exercise contexts and research more generally. Data richness was supported by in-depth focus group discussions (76–137 min), and the semi-structured, flexible, piloted, and open-ended questions enhanced data richness and minimized researcher influence. Accessibility was strengthened through reasonable adjustments and the use of both online and face-to-face focus groups, which broadened participation. Analytically, rigour was enhanced through inductive thematic analysis, independent coding with high intercoder agreement, and researcher reflexivity. The transparent reporting, in accordance with the APA JARS-Qual standards, further supports trustworthiness.

## Conclusion

This study explored service users’, instructors’, and strategic leaders’ perceptions and experiences of online exercise provision. The findings suggest that online exercise has considerable potential to complement in-person provision by offering a flexible and convenient mode of participation, while also facilitating opportunities for social connection. This may be particularly valuable for populations underserved by traditional services, for whom online exercise may act as a bridge to subsequent engagement in in-person exercise. However, effective implementation requires addressing technical, safety, privacy, and organizational challenges. Inclusive design, improving digital literacy, instructor training, organizational strategy, and cross-sector collaboration may facilitate its sustainable adoption.

## Data Availability

The anonymized transcripts are available via Figshare at: https://figshare.com/s/1685f690ea84a27c3fed.
